# Nefopam for the prevention of perioperative shivering: a meta-analysis of randomized controlled trials

**DOI:** 10.1186/s12871-015-0068-y

**Published:** 2015-06-09

**Authors:** Meng Lv, Xuetao Wang, Wendong Qu, Mengjie Liu, Yuelan Wang

**Affiliations:** 1 Department of anesthesiology, Qianfo shan Hospital, Shandong University, Jinan, Shandong China; 2 Department of anesthesiology, The Second affiliated hospital of Shandong Traditional Chinese Medicine University, Jinan, Shandong China; 3 Department of nosocomial infection management, the Central Hospital of Taian, Taian, Shandong China

**Keywords:** Nefopam, Shivering, Meta-analysis

## Abstract

**Background:**

Shivering is a frequent complication following surgery and anaesthesia. A large variety of studies have been reported that nefopam may be efficacious for the prevention and treatment of perioperative shivering. Regrettably, there is still no conclusion of the efficacy and safety of nefopam for the prevention of perioperative shivering. The aim of this analysis is to evaluate the efficacy of nefopam for the prevention of perioperative shivering in patients undergoing different types of anaesthesia compared with placebo group and other active interventions.

**Methods:**

PubMed, EMBASE, Cochrane Central Register of Control Trials were systematically searched for potentially relevant trials. Trial quality and extracted data were evaluated by two authors independently. Dichotomous data on the absence of shivering was extracted and analysed by using relative risk (RR) with 95 % confidence interval (CI). Continuous outcome was abstracted and analysed by using weighted mean difference (WMD) with 95 % confidence interval (CI). Outcome data was analysed by using random effect model or fixed effect model in accordance with heterogeneity.

**Results:**

Compared with placebo, prophylactic administration of nefopam significantly reduced the risk of perioperative shivering not only in the patients under general anaesthesia but also neuraxial anaesthesia (RR 0.08; 95 % CI 0.05-0.13). As compared with clonidine, nefopam was more efficacious in the prevention of perioperative shivering (RR 0.34; 95 % CI 0.17-0.70). Nefopam has no influence on the extubation time (WMD 0.92; 95 % CI −0.15-1.99).

**Conclusion:**

Our analysis has demonstrated that nefopam is associated with the decrease of risk of perioperative shivering following anaesthesia without influencing the extubation time.

## Background

Shivering is a distressing and frequent side effect during the intra-operative period [1], which might hinder monitoring [2]. Shivering never causes direct demise of patients and it is unlikely to become chronic. However, it is associated with the increase of oxygen consumption, which would endanger a patient with compromised cardiac function. The mechanism of perioperative shivering is not completely clear. Shivering may act as a response to hypothermia. Whereas, shivering happens even with normothermia in the perioperative period, suggesting that other origins than heat loss and subsequent decrease in body temperature may lead to the development of shivering [3]. Moreover, keeping patients normothermia is not always available. Thus, it is essential to prevent shivering by using pharmacologic interventions in a special case for selected patients.

A large variety of regimens have been demonstrated to be efficacious in the prevention and treatment of postanesthetic shivering, such as α2-agonist [4, 5], tramadol [1, 6], meperidine [7, 8], serotonin-3 receptor antagonist [9]. There is still no “gold standard” intervention for the prevention of perioperative shivering. To our knowledge, nefopam may hold the best promise due to its efficacy [10–12] without any serious adverse effects [13].

Nefopam, a benzoxazocine substance, is structurally related to orphenadrine and dyphehydramine [14]. Nefopam is neither a non-steroidal anti-inflammatory drug nor an opiate [15]. Nefopam is not associated with respiratory inhibition [16]. The mechanisms of nefopam involved in the anti-shivering and thermoregulatory action are not completely demonstrated. Nefopam inhibits the synaptic reuptake of dopamine, norepinephrine, and serotonin in an amphetamine fashion [17]. Nefopam affected thermoregulatory response via α_2_ adrenoceptors [18].

The aim of this review is to investigate the effectiveness of nefopam for preventing perioperative shivering and the influence of nefopam on the extubation time. The secondary aim of this analysis is to compare the efficacy of nefopam with the other active interventions for the prevention of perioperative shivering.

## Methods

We systematically screened the Medline (1966 - February 2015), EMBASE (1980 - February 2015), Cochrane Central Register of Controlled Trials (CENTRAL) (*The Cochrane Library 2015,* Issue 2) by using different search strategies. Potentially relevant randomised controlled trials (RCT) were searched by using search methods found in (Appendix 1 MEDLINE silver platter); (Appendix 2 EMBASE silver platter); (Appendix 3 CENTRAL). Grey literature and Conference proceedings were manually searched. Relevant investigators were contacted for supplemental data and special question. Additional articles were identified from reference lists of retrieved articles. No language restriction was applied in this study. The date of last search was February 18, 2015.

Randomised controlled trials of prophylactic administration of nefopam to prevent perioperative shivering were included in this analysis. Only standard randomised controlled trials were taken into account; quasi-randomized controlled studies, cluster-trials and cross-over trials were excluded. Active controlled trials without placebo group or inactive group were excluded. Studies with limited number of patients were excluded. Data from retrospective studies, nonsurgical settings studies or experimental studies in volunteers were not included. We only considered trials that investigated prophylactic use of nefopam for preventing perioperative shivering in adults undergoing surgery. Different doses of nefopam were tested in the included studies. We excluded the trials that were related to the treatment of shivering.

Each title and abstract of references was screened by two authors to exclude obvious irrelevant articles with disagreement resolved by consensus. If potentially relevant, the complete articles were retrieved. Two reviewers independently read the articles in complete form to determine the final inclusion. Consensus of inclusion of the studies was reached by discussion. Reviewers were not blinded to trial authors, institutions, journal of publication, and results of study.

The following inclusion criteria were used for potentially pertinent studies: random allocation, comparison of nefopam versus placebo, performed on surgical patients with no restraints on dose and timing of administration. The exclusion criteria were duplicate publications, nonhuman trials, without main outcome, studies in healthy volunteers and data from abstracts or letters.

Data were collected by two investigators from the selected studies, with disagreement resolved by consensus. We obtained the information on patients, anesthetic methods, interventions, outcomes, body temperature, different doses of nefopam and adverse effects.

For dichotomous outcome, data was extracted as the number of patients with outcome event and the number of patients in the treatment group and placebo group. A large variety of efficacy endpoints of shivering were reported in these studies. In order to reduce the interpretational bias, we only extracted efficacy data on complete absence of shivering. For continuous outcome, data was expressed as mean value and standard deviation (SD). Some of the included trials were multiple-arm studies. When we extracted the continuous data from these trials, it was necessary to combine multiple groups into a single one. The SD and mean values of the combined group were calculated according to the method of the Cochrane Handbook of Systematic Review of interventions [19] (Table 1).

**Table 1:** Formula for combining groups of continuous data

	Group 1	Group 2	Combined group
Sample size	N_1_	N_2_	N_1_ + N_2_
Mean	M_1_	M_2_	$\frac{N_1{M}_1+{N}_2{M}_2}{N_1+{N}_2}$
Standard Deviation	SD_1_	SD_2_	$\sqrt{\frac{\left({N}_1-1\right)S{D_1}^2+\left({N}_2-1\right)S{D_2}^2+\frac{N_1{N}_2}{N_1+{N}_2}\left({M_1}^2+{M_2}^2-2{M}_1{M}_2\right)}{N_1+{N}_2-1}}$

The risk of bias of the included studies was assessed by using the Cochrane Collaboration’s protocol [20]. The articles were reviewed to arrive a final score if there was a discrepancy.

Relative risk (RR) with pertinent 95 % confidence intervals (CI) were calculated for binary outcomes. A statistical significance between nefopam group and control group was assumed if the 95 % CI of the RR excluded 1. In the presence of significant heterogeneity, we intended to use random-effect method. If there was not statistically significant heterogeneity, the fixed effect method was used. As an estimate of statistically significant difference between nefopam and untreated control group for continuous variable, we calculated weighted mean difference (WMD) with 95 % CI. If the 95 % CI of WMD did not include 0, we considered that the difference between nefopam and control was significant. Heterogeneity and inconsistency were evaluated by Q and I^2^ test respectively. When the P value of Q test was greater than 0.10 and I^2^ was less than 50 %, we regarded it as homogeneity. The subgroup analysis was based on different anesthetic methods, general anaesthesia and neuraxial anaesthesia. Two sensitivity analyses were planned *a prior* to explore the heterogeneity. First, calculation that used the fixed effects model was repeated by using the random effect model. Second, some trials were retracted from the journal due to ethical reasons. Therefore, analysis was performed with or without these retracted articles. The reporting bias was assessed by visual inspection into funnel plots [21]. Meta-analysis was conducted by using Review Manager 5.1.

## Results

### Included studies

**Fig. 1:**
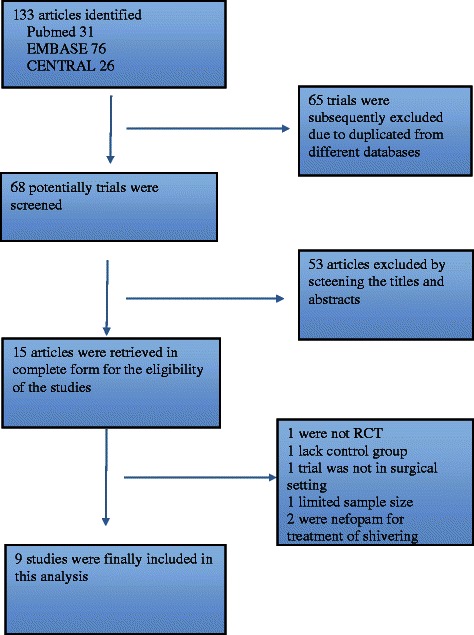
Flow chart of analyzed studies

Through electronic searches, snowballing, manual search, contact with the authors, 133 potentially eligible articles were obtained. The detail of flowchart of the studies included in this study was shown in Fig. 1.

A total of 124 studies were subsequently excluded. 65 reports were duplicated from different databases. 53 studies were obviously irrelevant trials of this analysis according to the titles and abstracts. One trial was not randomised controlled trials [22]. One trial lacked non-controlled trial or placebo group [7]. One trial was for the prevention of amphotericin-B induced shivering [23]. We excluded two studies of nefopam for the treatment of intra-operative shivering [24, 25]. Finally, in one trial sample size in each group less than 10 was excluded [26].

Nine eligible randomized clinical trials were eventually included in this analysis, which were published between 1992 and 2013 [13, 27–34] (Table 2). 925 participants from 9 studies were analysed in this study with 474 patients assigned to nefopam group, 131 patients allocated to clonidine group and 320 participants to placebo group. The sample size of enrolled studies ranged from 30 to 371. Six randomised clinical trials included in this analysis were undergoing general anaesthesia. The patients in two studies were undergoing neuraxial anaesthesia. In only one study, subjects were under conscious sedation. Six trials involved more than one active intervention or studied multiple experimental interventions. Of all trials nefopam was given intravenously. Nefopam was administered at different time points. In three studies nefopam was given before surgery. In 6 trials nefopam was administered at the end of surgery. Among all the enrolled studies, the participants in only one trial underwent active cooling. Three articles were retracted from the published journals due to ethical reason [29, 30, 35]. In five studies, sample size calculation was carried out to control, type one and type two errors.

**Table 2:** Demographic data of included studies

Study	Surgical setting	Type of anesthesia	Comparisons (No. of patients)	Time of administration	Temperature
Piper 2004 [29]	Orthopedic or abdominal	General anesthesia	Nefopam0.2 mg/kg (73); Nefopam0.1 mg/kg (75); Nefopam0.05 mg/kg (76); Clonidine1.5 μg/kg (73); Placebo (74)	At the end of surgery	Rectal temperature
Bilotta 2001 [33]	Craniotomy	General anesthesia	Nefopam0.12 mg/kg (20); Placebo (20)	At the end of surgery	Bladder temperature
Piper 1999 [30]	Abdominal or orthopedic	General anesthesia	Nefopam0.15 mg/kg (20); Clonidine3μg/kg (20); placebo (20)	At the end of surgery	Rectal temperature
Bilotta 2002 [13]	Lower limb orthopedic	Neuraxial anesthesia	Nefopam0.15 mg/kg (30); Tramadol0.5 mg/kg (30); Placebo (30)	Before anesthesia	Urinary-bladder temperature
Bilotta 2005 [28]	Interventional neuroradiology	Conscious sedation	Nefopam0.15 mg/kg (32); Clonidine3μg/kg (38); Placebo (31)	Before anesthesia	Bladder temperature
Rohm 2005 [27]	Abdominal or urological	General anesthesia	Physostigmine2mg (31); Nefopam10mg (30); Placebo (28)	At the end of surgery	Tympanic temperature
Kamal 2011 [34]	Orthopedic	Neuraxial anesthesia	Nefopam0.2 mg/kg (76); Ketamine + midazolam (75); placebo (75)	Before anesthesia	Tympanic temperature
Piper 1998 [31]	Orthopedic or abdominal	General anesthesia	Nefopam0.15 mg/kg (15); Placebo (15)	At the end of surgery	Rectal temperature
Tempia 1992 [32]	Radical cystectomy	General anesthesia	Nefopam (27); placebo (27)	At the end of surgery	Not mentioned

### Risk of bias in included studies

**Fig. 2:**
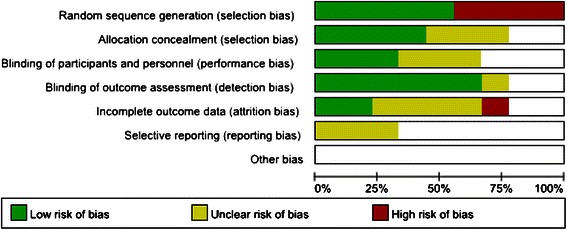
Risk of bias graph

The quality assessment of the included studies was based on: method of randomization, concealment of allocation, use of blinding, and completeness of data. The detail of the risk of bias was presented in Fig. 2. The funnel plot was presented in Fig. 4 and there was not evident publication bias.

**Fig. 3:**
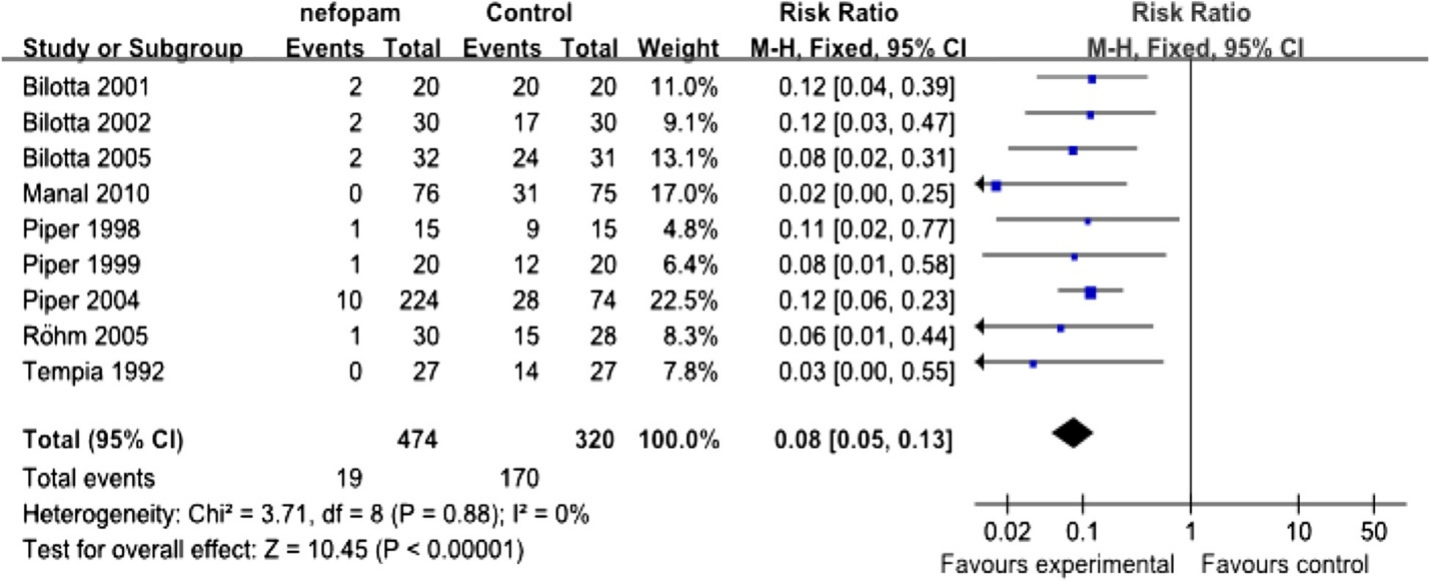
The effect of nefopam on the perioperative shivering

**Fig. 4:**
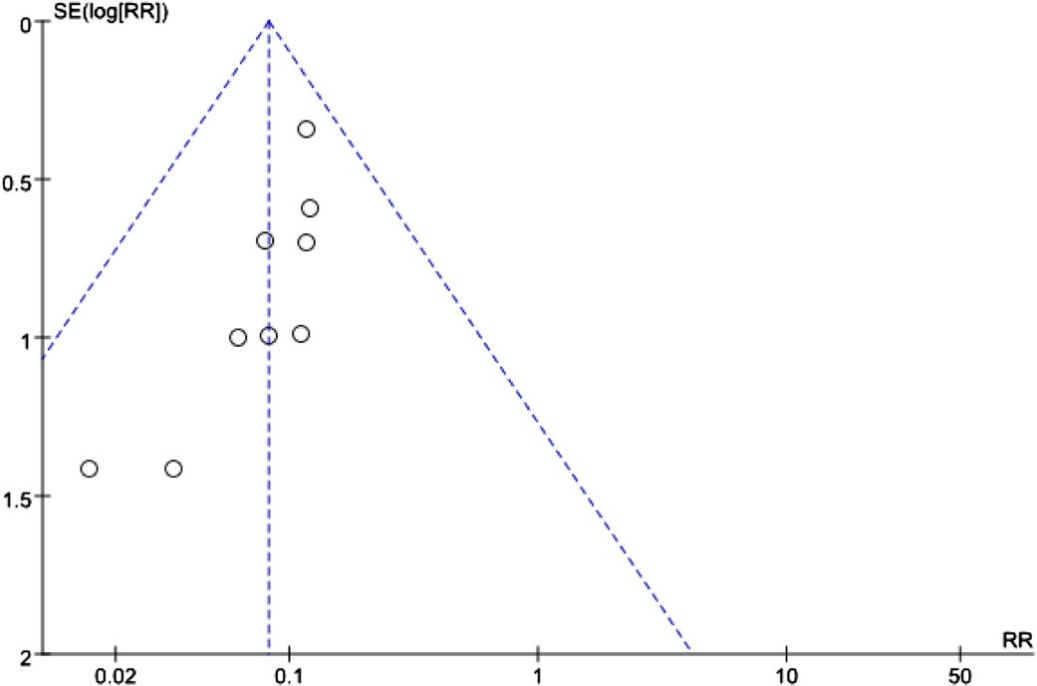
Funnel plots for the influence of nefopam on the incidence of peri-operative shivering

**Fig. 5:**
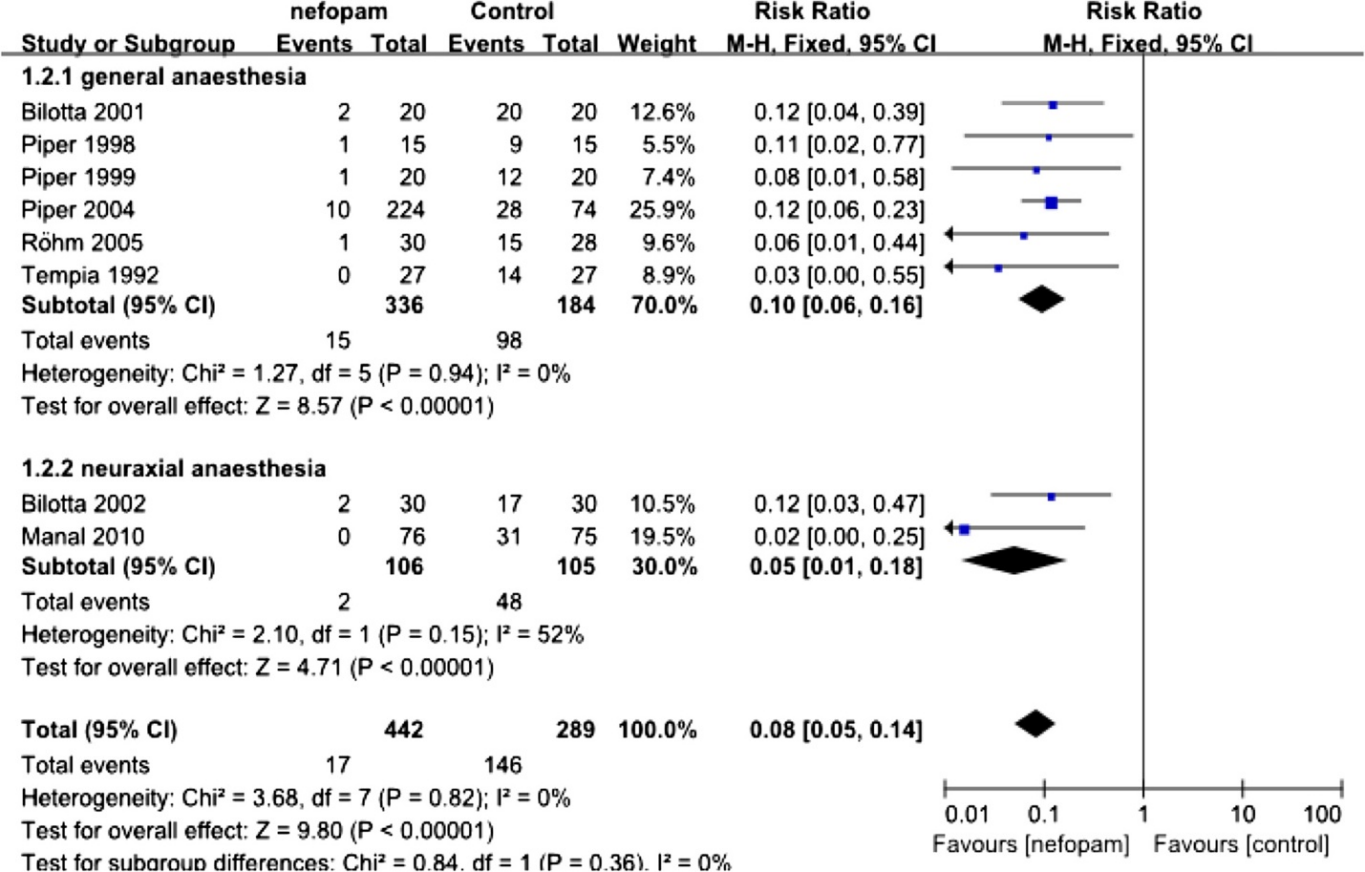
The effect of nefopam on perioperative shivering under different anesthesia

**Fig. 6:**
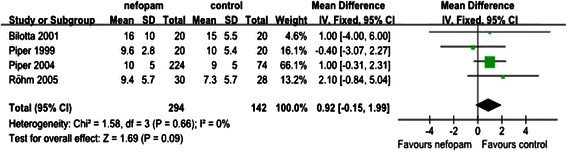
The infuence of nefopam on the extubation time

**Fig. 7:**
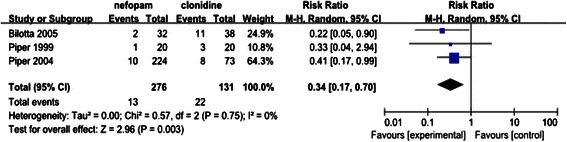
The influence of nefopam on the peri-operative shivering in comparison with clonidine

### Effects of intervention

#### Risk of perioperative shivering

9 studies reporting on the episodes of shivering finally entered into the analysis, with 474 patients allocated to nefopam group and 320 patients to placebo group. The control group event rate ranged from 16.7 % to 100 %. Overall analysis demonstrated that nefopam was associated with significant reduction in the incidence of shivering (RR,0.08, 95 % CI 0.05-0.13)as shown in Fig. 3. Statistical tests indicated that there was no substantial heterogeneity across the studies (Chi^2^ = 3.71, *P* = 0.88, I^2^ = 0 %), therefore data on the incidence of shivering was combined by using the fixed effect model.

#### Sensitivity analysis

1. We applied two sensitivity analyses to the incidence of shivering compared nefopam with placebo.
  The fixed effect model favoured prophylactic use of nefopam (RR 0.08, 95 % CI 0.05- 0.13).
  The random effect model also favoured the administration of nefopam (RR 0.10, 95 % CI 0.06-0.15).

2 There were three articles retracted from published journals due to ethnic reasons. This sensitivity analysis considered the retracted studies.
  The incidence of shivering was significantly lower in the nefopam group than the placebo group without the retracted articles (RR 0.09, 95 % CI 0.05- 0.17).

#### Subgroup analysis

Subgroup analysis focused on the different methods of anaesthesia, general anaesthesia and neuraxial anaesthesia, as shown in Fig. 5.

General anaesthesia: the RR favoured administration of nefopam (RR 0.10, 95 % CI 0.06, 0.16).

Neuraxial anaesthesia: the RR favoured prophylactic administration of nefopam (RR 0.05, 95 % CI 0.01, 0.18).

#### The effect of nefopam on extubation time

Four studies tested on the effect of nefopam on extubation time compared to the control group. When the data was combined, the extubation time in placebo group was earlier than nefopam group. However, the difference between the two groups did not reach statistical significance (WMD0.92; 95 % CI −0.15, 1.99), the data was presented in Fig. 6.

#### Comparison of nefopam with clonidine

Clonidine was the most frequently tested substance. Three studies reported on the risk of shivering comparing nefopam with clonidine in total of 407 patients, with 276 allocated to nefopam group and 131 to clonidine group. When the data was combined, the risk of perioperative shivering was significantly lowered in patients received nefopam in comparison with clonidine (RR, 0.34, 95 % C I0.17-0.70). There was no statistical heterogeneity between nefopam group and clonidine group (Chi^2^ = 0.57, *P* = 0.75, I^2^ = 0 %), as shown in Fig. 7.

## Discussion

We performed a meta-analysis following the guideline of methodology of the Cochrane Handbook for systemic review of interventions. There are two main results in this research. First, this analysis revealed that nefopam was associated with the reduction of rate of shivering not only under general anaesthesia but also neuraxial anaesthesia. As compared with clonidine, nefopam was more efficacious for the prevention of postanaesthetic shivering during the perioperative period. Second, as compared with placebo group, the extubation time in nefopam group did not show statistically significant difference. Besides, none of the enrolled studies reported on any serious side effect of nefopam, such as respiratory depression or cardiovascular effects.

To our knowledge, this is the first meta-analysis of nefopam for preventing perioperative shivering. The methodological quality of most of the included studies was of high quality. There was a large sample size in this analysis, so it was possible to determine the significant difference between the nefopam group and control group. As a comprehensive search has been done by our research group, it is nearly impossible that any important evidence has been ignored.

The aetiology of perioperative shivering is related to hypothermia, decreased sympathetic activity, pyrogen release, postoperative pain, adrenal suppression, depression of vasoconstriction following anaesthesia, blood loss, duration of surgery et al., [36] it has to be kept in mind that maintaining body core temperature have a major effect on the risk of perioperative shivering. Nevertheless, we intentionally kept the patients hypothermic [37] sometimes and it is impossible to keep the body core temperature within normal range all the time. Moreover, shivering occurred even in normothermic patients [38]. Therefore, simple and inexpensive pharmacological strategy is encouraged by anesthesiologists.

A large variety of pharmacological regimens were proposed for the treatment or prevention of shivering. Most of these interventions are efficacious for perioperitive shivering. From the indirect comparison of previous Meta-analysis, nefopam was more efficacious than other interventions [10–12]. This is consistent with our analysis that nefopam was more efficacious than clonidine from the direct comparisons of the included trials. Notwithstanding, there were significantly clinical and statistical heterogeneity among the included studies and report of direct comparison was sparse.

Among all the pharmacological interventions, clonidine was the most frequently reported drug. According to the previous randomized controlled trials and meta-analysis, clonidine was effective in the treatment and prevention of postanesthetic shivering. However, clonidine was associated with some side effects, for instance, sedative effect, hypotension and bradycardia. Thus, these side effects limited its use. The opioids, such as tramadol, meperidine, alfentanil were also effective in the prevention and treatment of shivering. Nonetheless, these drugs were related to the risk of respiratory depression and postoperative nausea and vomiting (PONV). According to all of the enrolled studies, nefopam was not associated with the increase of the risk of any serious side effect, which contribute much to its safety. None of the included studies reported on any side effect of nefopam. This is inconsistent with the previous reports [7, 39, 40]. This is probably because the subjects of most of the included studies were under general anaesthesia.

It is encouraging that nefopam may possess a specific shivering effect. Alfonsi et al. has shown that nefopam caused a small increase in the core temperature by lowering the shivering threshold and without influencing sweating and vasoconstriction thresholds, therefore minimizing heat loss [41]. Whereas, the other antishivering drugs, such as clonidine [42], meperidine [43], volatiles reduce both shivering and vascular threshold [44], tamadol [45], thus leading to greater heat loss.

There are several limitations in our analysis. Firstly, the methods of reporting the scores of perioperative shivering were different between the trials in this analysis, most of the included studies used Wrench et al. evaluation [46], meanwhile, some trials used their own scoring systems. In this analysis, we only abstracted binary data on absence of shivering to minimise the interpretational risk. Secondly, the sample size of some trials was limited. Small sample size may overestimate the efficacy of nefopam in the prevention of shivering compared with large trial. Thirdly, the baseline of control event rate has a large variability. With extremely frequent control event rate, it may exaggerate the intervention’s performance. The patients whom anesthesiologists see in daily clinical work do not have such a high frequent incidence of shivering. This phenomenon challenges the external clinical applicability of this study. Fourthly, reports of the adverse effects were sparse. However, lack of reports does not represent that none occurred. None of the included studies reported on the injection pain, probably because most of the included studies were under general anaesthesia. This was not in agreement with previous study reporting high incidence of injection pain. Finally, in this meta-analysis, reviewers were not blinded to study authors, which might increase the risk of bias. In the future study, investigators should be blinded to study authors, institutions, journal of publication, and results of study.

## Conclusions

In conclusion, we have demonstrated that nefopam inhibit perioperative shivering without producing hypotension and sedative of α-2 agonist and respiratory influence of opiates. The better prophylactic antishivering efficacy of nefopam and the side effects of other interventions suggest that among all the interventions, nefopam may hold the best promise. However, side effects of nefopam need to be investigated. Besides, direct comparison of nefopam with other drugs, such as tramadol, meperidine is stillke needed to confirm the relative efficacy.

## Appendix

### Appendix 1 MEDLINE search strategy

#1 nefopam

#2 “nefopam” [Mesh]

#3 #1 OR #2

#4 shivering

#5”shivering” [Mesh]

#6 #4 OR #5

#7 #3 AND #6

#8 #7 Filters Randomized controlled trial

### Appendix 2 EMBASE search strategy

#1 nefopam

#2 “nefopam” [EMTREE]

#3 #1 OR #2

#4 shivering

#5 “shivering” [EMTREE]

#6 #4 OR #5

#7 #3 AND #6

#8 #7 Filters Randomized controlled trial

### Appendix 3 Search strategy for CENTRAL, The Cochrane Library

#1 MeSH descriptor nefopam explode all trees

#2 nefopam

#3 #1 OR #2

#4 MeSH descriptor shivering explode all trees

#5 shivering

#6 #4 OR #5

#7 #3 AND #6

## Abbreviations

PONV: Postoperative nausea and vomiting
WMD: Weighted mean difference
RR: Relative risk
SD: Standard difference
CI: Confidence interval
